# Pushing the Limits of EEG: Estimation of Large-Scale Functional Brain Networks and Their Dynamics Validated by Simultaneous fMRI

**DOI:** 10.3389/fnins.2020.00323

**Published:** 2020-04-16

**Authors:** Rodolfo Abreu, Marco Simões, Miguel Castelo-Branco

**Affiliations:** ^1^Faculty of Medicine, Coimbra Institute for Biomedical Imaging and Translational Research (CIBIT), Institute for Nuclear Sciences Applied to Health (ICNAS), University of Coimbra, Coimbra, Portugal; ^2^Center for Informatics and Systems (CISUC), University of Coimbra, Coimbra, Portugal

**Keywords:** simultaneous EEG-fMRI, large-scale functional brain networks, dynamic functional connectivity (dFNC), electrical source imaging (ESI), task-based fMRI, resting-state functional network connectivity (rs-FNC)

## Abstract

Functional magnetic resonance imaging (fMRI) is the technique of choice for detecting large-scale functional brain networks and to investigate their dynamics. Because fMRI measures brain activity indirectly, electroencephalography (EEG) has been recently considered a feasible tool for detecting such networks, particularly the resting-state networks (RSNs). However, a truly unbiased validation of such claims is still missing, which can only be accomplished by using simultaneously acquired EEG and fMRI data, due to the spontaneous nature of the activity underlying the RSNs. Additionally, EEG is still poorly explored for the purpose of mapping task-specific networks, and no studies so far have been focused on investigating networks’ dynamic functional connectivity (dFC) with EEG. Here, we started by validating RSNs derived from the continuous reconstruction of EEG sources by directly comparing them with those derived from simultaneous fMRI data of 10 healthy participants, and obtaining an average overlap (quantified by the Dice coefficient) of 0.4. We also showed the ability of EEG to map the facial expressions processing network (FEPN), highlighting regions near the posterior superior temporal sulcus, where the FEPN is anchored. Then, we measured the dFC using EEG for the first time in this context, estimated dFC brain states using dictionary learning, and compared such states with those obtained from the fMRI. We found a statistically significant match between fMRI and EEG dFC states, and determined the existence of two matched dFC states which contribution over time was associated with the brain activity at the FEPN, showing that the dynamics of FEPN can be captured by both fMRI and EEG. Our results push the limits of EEG toward being used as a brain imaging tool, while supporting the growing literature on EEG correlates of (dynamic) functional connectivity measured with fMRI, and providing novel insights into the coupling mechanisms underlying the two imaging techniques.

## Introduction

A large-scale functional brain network is defined as a subset of interconnected, possibly distant, brain regions that interact with each other in order to perform a plethora of tasks of different levels of complexity (Bressler and Menon, 2010). The identification of such networks led to pivotal findings regarding brain function in healthy humans (van den Heuvel and Hulshoff Pol, 2010), and by discriminating changes in some properties of those networks due to disease, a better understanding of their pathophysiology was possible (Du et al., 2018). Because of its remarkable spatial resolution and whole-brain coverage, functional magnetic resonance imaging (fMRI) is currently the imaging technique of choice to measure the connectivity strength (or functional connectivity, FC) between brain regions, and consequently reconstruct such functional networks with a few millimeter resolution, despite its modest temporal resolution in the order of seconds (van den Heuvel and Hulshoff Pol, 2010; Lee et al., 2013). While task-based fMRI studies have allowed to map the brain regions that specifically respond to the task of interest (Barch et al., 2013), several large-scale functional networks can be found in the normal brain during rest – the so-called resting-state networks (RSNs) – which exhibit temporally correlated spontaneous fluctuations in the blood-oxygen-level-dependent (BOLD) signal (Biswal et al., 1995; Smith et al., 2009). RSNs are typically identified by decomposing the fMRI data using spatial independent component analysis (sICA), under the hypothesis that the resulting independent components (including the RSNs) are statistically independent in space (Beckmann et al., 2005).

Importantly, RSNs are also identified under the assumption that their functional connectivity is static; however, current literature suggests that brain networks in general continuously reorganize in response to both internal and external stimuli at multiple time-scales, resulting in temporal fluctuations of their FC – the so-called dynamic functional connectivity (dFC) (Hutchison et al., 2013; Calhoun et al., 2014; Preti et al., 2017). Specifically, it has been shown that dFC correlates with brain state (stimulation/task, eyes closure vs. eyes open, vigilance, sleep, anesthesia, and drug manipulation) as well as with age, gender and disease [for a review, please refer to Thompson (2018)]. dFC correlates of disease are believed to be particularly relevant for its characterization, and recent studies have demonstrated that dFC discriminates healthy from patient populations better than static FC (Du et al., 2018). Under the assumption that brain function dynamics can be described by a limited number of states (Preti et al., 2017), a significant number of studies have dedicated to the identification of such brain states from the dFC, by applying pattern recognition techniques, particularly clustering (Allen et al., 2014), principal component analysis (Leonardi et al., 2013), and dictionary learning (Leonardi et al., 2014; Abreu et al., 2019).

Despite the undoubted insights that the study of brain networks’ (dynamic) functional connectivity with fMRI has provided so far, this imaging technique is only capable of indirectly measuring brain activity/connectivity, delayed by the hemodynamic response (Logothetis et al., 2001). In contrast, magnetoencephalography (MEG) and electroencephalography (EEG) measure the activity of large populations of neurons directly, and because of their high temporal resolution at the sub-millisecond scale, they represent in principle ideal approaches to study brain functional connectivity (Niedermeyer and Lopes Da Silva, 2005). Whole-brain FC studies using MEG or EEG, however, require the challenging procedure of reconstructing the sources responsible for generating the signals measured at the scalp (Mantini et al., 2011; Michel and Murray, 2012). Due to continuous technological advances, a reliable reconstruction of MEG and EEG is now possible, opening the pathway for the study of large-scale FC with high temporal resolution. In fact, RSNs typically identified with fMRI were first replicated on MEG (Brookes et al., 2011; Mantini et al., 2011), and more recently on EEG (Liu et al., 2017, 2018), by first performing continuous electrical source imaging (cESI), which reconstructs their underlying sources over time (Vulliemoz et al., 2010a; Michel and Murray, 2012), and then applying sICA to the resulting dataset. This suggests that temporally coherent fluctuations across distant brain regions can also be captured with MEG and EEG. Motivated by its portability, low cost and ease of use (Niedermeyer and Lopes Da Silva, 2005), and more importantly, the possibility of combining it with other imaging modalities, particularly fMRI (Abreu et al., 2018), EEG is frequently preferred over MEG.

Because reconstructing EEG sources involves a complex pipeline (Michel et al., 2004; Michel and Murray, 2012), a recent study has systematically investigated the impact of the several processing steps on the accurate identification of RSNs with EEG during rest (Liu et al., 2018). However, the ground truth considered was based on RSNs derived from separately acquired fMRI data; additionally, the feasibility of using EEG data to map task-specific brain networks has been poorly explored, and the estimation of dFC and the associated brain states with EEG is yet to be investigated. Because RSNs have been shown to be also present in task-based fMRI studies (Di et al., 2013; Cole et al., 2016), in this paper we start by validating in a truly unbiased manner the results from Liu et al. (2017, 2018) by identifying RSNs on fMRI and EEG data acquired simultaneously from 10 healthy participants performing a neurofeedback (NF) task. Next, we mapped the target region of the NF (the facial expressions processing network, FEPN) with fMRI and EEG, and compared the resulting networks. Finally, we estimated dFC brain states also from fMRI and EEG, and determined into which extent these techniques match.

## Materials and Methods

### Participants

Ten healthy participants (mean age: 26 ± 3 years; 9 males) performed a simultaneous EEG-fMRI NF session. All participants had normal or corrected-to-normal vision, and no history of neurological disorders. The study was approved by the Ethics Commission of the Faculty of Medicine of the University of Coimbra and was conducted in accordance with the declaration of Helsinki. All subjects provided written informed consent to participate in the study.

### Experimental Protocol

The session was performed at the Portuguese Brain Imaging Network (Coimbra, Portugal) and consisted of four simultaneous EEG-fMRI runs: first, a functional localizer specifically developed to identify the FEPN (anchored on the posterior Superior Temporal Sulcus region; pSTS), followed by three NF runs (of alternated up and down regulation). The first two NF runs used either visual or auditory feedback (random order) and the third had no feedback presented to the participant. The participants were given a mental strategy to follow during the NF runs, but were instructed to adapt it to maximize the modulation outcomes presented by the feedback.

For the localizer run, 8 s blocks of dynamic facial expressions (happy, sad or alternated between happy and sad expressions) morphed into the face of a realistic human virtual character are contrasted with 8 s blocks consisting of the same face with a static neutral expression, or motion blocks of moving dots. This rigorous contrast is able to identify regions that respond to the dynamic aspects of the facial expressions but not to the face itself or the movement aspects of the stimuli (Direito et al., 2019). The NF runs consist of 24 s blocks of alternated up and down regulation of the activity extracted in real-time from the pSTS region identified in the localizer run. For the first two NF runs, the participants were presented with visual feedback (materialized in the intensity level of the facial expression displayed by the virtual avatar) or auditory feedback (consisting of high and low pitch beep sounds, corresponding to increasing or decreasing the amplitude of the BOLD signal in the pSTS region, respectively). A detailed description of the protocol can be found in the Supplementary Material, and in our previous paper (Direito et al., 2019), where we assessed the neuroscientific aspects of the fMRI NF sessions.

### EEG-fMRI Data Acquisition

Imaging was performed on a 3T Siemens Magnetom Trio MRI scanner (Siemens, Erlangen) using a 12-channel RF receive coil. The functional images were acquired using a 2D multi-slice gradient-echo echo-planar imaging (GE-EPI) sequence, with the following parameters: TR/TE = 2000/30 ms, voxel size = 4.0 mm × 4.0 mm × 3.0 mm, 33 axial slices, FOV = 256 mm × 256 mm, FA = 90°, yielding a total coverage of the occipital and posterior temporal lobe. The start of each trial was synchronized with the acquisition of the functional images. A T_1_-weighted, magnetization-prepared rapid acquisition gradient-echo (MPRAGE) sequence was used for the acquisition of structural data (1 mm isotropic, 176 slices, TR/TE = 2530/3.42 ms), allowing for the subsequent co-registration of the functional data. For each participant, 160 fMRI volumes were acquired during the localizer run, yielding approximately 5.33 min of duration; the remaining three functional (NF) runs consisted of 300 volumes (10 min).

The EEG signal was recorded using the MR-compatible 64-channel NeuroScan SynAmps2 system and the Maglink software, with a cap containing 64 Ag/AgCl non-magnetic electrodes positioned according to the 10/10 coordinate system, a dedicated electrode for referencing placed close to the Cz position, and two electrodes placed on the chest for electrocardiogram (EKG) recording. EEG, EKG, and fMRI data were acquired simultaneously in a continuous way, and synchronized by means of a Syncbox (NordicNeuroLab, United States) device. EEG and EKG signals were recorded at a sampling rate of 10 kHz. No filters were applied during the recordings. The helium cooling system was not turned off, as it may carry the associated risk of helium boil-off in certain systems (Mullinger et al., 2008), and thus is not permitted in some clinical sites as the one used in this study.

Outside the MR scanner and prior to the beginning of the experiment, each subject was submitted to an EEG-only acquisition while performing the localizer stimulation experiment. These data were used to check the quality of the EEG recorded inside the MR scanner, after the removal of gradient switching and pulse artifacts.

All the procedures described next were coded in MATLAB, and are available together with all data upon request.

### Data Pre-processing

#### Electroencephalography (EEG)

##### Removal of EEG artifacts

EEG data underwent gradient artifact correction on a volume-wise basis using a standard artifact template subtraction (AAS) approach (Allen et al., 2000). Then, bad epochs of 2 s (corresponding to one TR) were manually identified and removed from the EEG signal; these were selected based on EEG segments of abnormally high amplitude and/or frequency, or whenever the gradient artifact correction algorithm failed to remove the artifact, typically in the beginning and ending of the recordings. Bad channels were visually inspected and interpolated. For the removal of the pulse artifact, the method presented in Abreu et al. (2016) was employed, whereby the EEG data are first decomposed using independent component analysis (ICA), followed by AAS to remove the artifact occurrences from the independent components (ICs) associated with the artifact. The corrected EEG data are then obtained by reconstructing the signal using the artifact-corrected ICs together with the original non-artifact-related ICs. Finally, the EEG data were down-sampled to 250 Hz and band-pass filtered to 1–45 Hz. For the purpose of removing the pulse artifact from the EEG, and the physiological noise from the fMRI (described below), the Pan-Tompkins algorithm (Pan and Tompkins, 1985) was optimized and used for the detection of R peaks on the EKG data (Abreu et al., 2017).

##### Continuous EEG source imaging

The pre-processed EEG data were then submitted to an EEG source imaging (ESI) procedure. Specifically, the so-called continuous ESI was performed, with the purpose of estimating the temporal variations of the EEG sources responsible for generating the electrical potential distributions measured at the scalp with a high temporal resolution. A realistic head model was built by first segmenting each subject’s structural image into 12 tissue classes (skin, eyes, muscle, fat, spongy bone, compact bone, cortical gray matter, cerebellar gray matter, cortical white matter, cerebellar white matter, cerebrospinal fluid, and brain stem), each with a specific electrical conductivity [depicted in Supplementary Table S1; from Liu et al. (2017)]. Because this is not feasible using the currently available tools (which allow segmentation up to six tissues), a state-of-the-art brain tissue model was used (MIDA, available online; Iacono et al., 2015), and co-registered into the structural image using FSL’s tool FNIRT (Jenkinson and Smith, 2001; Jenkinson et al., 2002). The transformed MIDA model onto the structural image of each participant was then visually inspected for assessing the quality of the registration step. The electrode positions were co-registered to the skin compartment by first considering their standard positions, and then manually adjusting them to match the distortions clearly observed on the structural images. Finally, the SimBio finite element model (FEM; Wolters et al., 2004) algorithm implemented in FieldTrip (Oostenveld et al., 2011) was then used for numerically approximating the volume conduction model (the number of FEM elements in each tissue compartment is depicted in Supplementary Table S1). The source dipoles were placed on a 4-mm 3D grid only spanning the cortical gray matter, followed by the estimation of the leadfield matrix, which maps each possible dipole configuration onto a scalp potential distribution (the forward problem). The inverse problem was solved through a distributed inversion solution approach using the exact low resolution brain electromagnetic tomography (eLORETA; Pascual-Marqui et al., 2011) algorithm, which estimates the strength *j* of each dipole on the source grid at the *x* (*j*_x_), *y* (*j*_y_), and *z* (*j*_z_) directions. For each EEG time sample *t*, the overall strength of a dipole was computed as:

(1) $$p\,\left(t\right)=\sqrt{j_{\text{x}}^{2}\,\left(t\right)+j_{\text{y}}^{2}\,\left(t\right)+j_{\text{z}}^{2}\,\left(t\right)}$$

This was computed for all dipoles and time samples, yielding a 4D (3D × *t*) dataset (EEG-ESI). The time-series of dipole strength was then downsampled to 1 Hz. These processing steps were selected based on previous studies that comprehensively investigated their impact on detecting RSNs from EEG-ESI data, concluding that this was the optimal processing pipeline (Liu et al., 2017, 2018).

#### Functional Magnetic Resonance Imaging (fMRI)

The first 10 s of data were discarded to allow the BOLD-fMRI signal to reach steady-state, and non-brain tissue was removed using FSL’s tool BET (Smith, 2002). Subsequently, slice timing and motion correction were performed using FSL’s tool MCFLIRT (Jenkinson et al., 2002). Then, a high-pass temporal filtering with a cut-off period of 100 s was applied, and spatial smoothing using a Gaussian kernel with full width at half-maximum (FWHM) of 5 mm was performed. Physiological noise was removed by linear regression using the following regressors (Abreu et al., 2017): (1) quasi-periodic BOLD fluctuations related to cardiac cycles were modeled by a fourth order Fourier series using RETROICOR (Glover et al., 2000); (2) aperiodic BOLD fluctuations associated with changes in the heart rate were modeled by convolution with the respective impulse response function [as described in Chang et al. (2009)]; (3) the average BOLD fluctuations in white matter (WM) and cerebrospinal fluid (CSF); (4) the six motion parameters estimated by MCFLIRT; and (5) scan nulling regressors (motion scrubbing) associated with volumes acquired during periods of large head motion (12 ± 7 scrubbed volumes, averaged across runs and subjects); these were determined using the FSL’s utility *fsl_motion_outliers*, whereby the DVARS metric proposed in Power et al. (2012) is first computed, and then thresholded at the 75th percentile plus 1.5 times the inter-quartile range. Because the respiratory traces were not recorded, the associated physiological fluctuations in the BOLD signal were not removed. Finally, volumes corresponding to bad epochs identified on the EEG were removed.

For each participant, WM and CSF masks were obtained from the respective T_1_-weighted structural image by segmentation into gray matter, WM and CSF using FSL’s tool FAST (Zhang et al., 2001). The functional images were co-registered with the respective T_1_-weighted structural images using FSL’s tool FLIRT, and subsequently with the Montreal Neurological Institute (MNI) (Collins et al., 1994) template, using FSL’s tool FNIRT (Jenkinson and Smith, 2001; Jenkinson et al., 2002). Both WM and CSF masks were transformed into functional space and were then eroded using a 3 mm spherical kernel in order to minimize partial volume effects (Jo et al., 2010). Additionally, the eroded CSF mask was intersected with a mask of the large ventricles from the MNI space, following the rationale described in Chang and Glover (2009).

### Data Analysis

In this section, the several data analyses performed on both fMRI and EEG-ESI for the identification of RSNs, the FEPN and the dFC brain states are described. Except when stated otherwise, the same procedures were applied to the fMRI and EEG-ESI data.

#### Identification of Resting-State Networks (RSNs)

##### ICA decomposition

Following the approach described in Liu et al. (2017, 2018), the pre-processed fMRI and EEG-ESI data were submitted to a group-level probabilistic spatial ICA (sICA) decomposition using the FSL’s tool MELODIC (Beckmann and Smith, 2004), whereby the data of all participants for each run are temporally concatenated prior to the sICA step, as recommended in the MELODIC’s guide for the identification of RSNs^1^. The optimal number of independent components (ICs) was automatically estimated for the fMRI data based on the eigenspectrum of its covariance matrix (Beckmann and Smith, 2004), with an average of approximately 40 ICs across runs. Because the dimensionality estimation algorithm in MELODIC is tailored for fMRI data, and for consistency purposes, the EEG-ESI data were also decomposed into 40 ICs. Nonetheless, when applying this algorithm to the EEG-ESI data, a similar number of ICs was estimated, differing no more than five ICs across runs with relation to those estimated from the fMRI data.

##### Automatic identification of RSNs

An automatic procedure for the identification of well-known RSNs was then applied, in which the spatial maps of the ICs (thresholded at *Z* = 3.0) were compared with those of the 10 RSN templates described in Smith et al. (2009), in terms of spatial overlap computed as the Dice coefficient (Dice, 1945). For each template, the IC map yielding the highest Dice coefficient was determined as the corresponding RSN. In the cases of non-mutually exclusive assignments, the optimal assignment was determined by randomizing the order of the RSN templates (up to a maximum of 10,000 possible combinations were considered, for computational purposes), and then sequentially, and mutually exclusively, assigning them to the IC maps based on their Dice coefficient. The assignment with the highest average Dice coefficient across all RSN templates was then deemed optimal, yielding the final set of RSNs: three visual networks (RSN 1–3), the default mode network (DMN and RSN4), a cerebellum network (RSN5), a motor network (RSN6), an auditory network (RSN7), the salience network (RSN8), a right language network (RSN9), and a left language network (RSN10). Although the RSN templates serve as an independent validation of the RSNs, the Dice coefficient between fMRI and EEG-ESI RSNs was also computed, thus allowing to directly compare our results with the current literature (Liu et al., 2017, 2018).

##### Statistical validation

In order to statistically validate the abovementioned spatial overlaps, a null model based on the concept of commonly used permutation tests was defined. Specifically, since it was hypothesized that 10 RSNs could be unequivocally identified among the 40 fMRI and EEG-ESI ICs (based on their substantially higher overlap with RSN templates when compared with other ICs unrelated with RSNs), the null model was obtained from the Dice coefficients computed for all the possible combinations of RSN templates and fMRI/EEG-ESI ICs (a total of 40 [ICs] × 10 [RSNs] = 400 values). In this way, for each run, null distributions of Dice coefficients were defined for: (1) the overlap between RSN templates and fMRI ICs; (2) the overlap between RSN templates and EEG-ESI ICs; and (3) the overlap between fMRI and EEG-ESI ICs. By computing their associated 95th percentile, a statistical threshold was then determined for each analysis, against which the average Dice coefficients across RSNs were tested (values in Table 1).

**TABLE 1:** Dice coefficients between the fMRI and EEG RSNs and RSN templates from Smith et al. (2009), and between the RSNs derived from fMRI and EEG-ESI data, for each run separately.

**Functional runs**	**fMRI RSNs vs. RSN templates**		**EEG RSNs vs. RSN templates**		**fMRI RSNs vs. EEG RSNs**	
	*d*_max_	$\bar{d}$	*d*_max_	$\bar{d}$	*d*_max_	$\bar{d}$
Auditory feedback	0.7	0.5	0.6	0.4	0.5	0.4
Visual feedback	0.7	0.5	0.6	0.4	0.5	0.3
Transfer run	0.7	0.5	0.6	0.4	0.5	0.4
Localizer	0.6	0.5	0.5	0.4	0.7	0.4

#### Mapping of the Facial Expressions Processing Network

For the purpose of mapping the regions in the FEPN, a general linear model (GLM) framework was used on both fMRI and EEG-ESI data. Although uncommon, the rationale of also using a GLM to analyze EEG data followed that of previous studies (Custo et al., 2014; Gonçalves et al., 2014), including the LIMO EEG toolbox which was specifically designed under this framework (Pernet et al., 2011). For the localizer run of each participant, a GLM comprising five regressors was built (one for each condition; described in section “Experimental Protocol”), based on unit boxcar functions with ones during the respective condition to be modeled, and zeros elsewhere. While this GLM was used directly to model the activity of the FEPN on the EEG-ESI data since no hemodynamic delay is expected, for the fMRI data, the regressors were convolved with a canonical, double-gamma hemodynamic response function (HRF) to account for such delay (Friston et al., 1995). The respective GLMs were then fitted to the pre-processed fMRI and the EEG-ESI data using the FSL’s improved linear model (FILM) (Woolrich et al., 2001), and voxels exhibiting significant signal changes when contrasting the facial expression conditions with the neutral and motion conditions (balanced) were identified by cluster thresholding (voxel *Z* > 2.7, cluster *p* < 0.05). Group activation maps were then obtained using the FSL’s Local Analysis of Mixed Effects (FLAME) (Beckmann et al., 2003).

#### Dynamic Functional Connectivity Analysis

##### Estimation of dFC

For each run, the dFC was estimated between *R* = 90 non-overlapping regions of interest (ROIs) of the cerebrum according to the automated anatomical labeling (AAL) atlas (Tzourio-Mazoyer et al., 2002). These ROIs were co-registered from the MNI space into the participant’s functional space, and the EEG-ESI and the pre-processed fMRI data were then averaged within each ROI. Regarding the latter, the resulting BOLD signals were low-pass filtered with a cutoff frequency of 0.1 Hz, because synchronized BOLD fluctuations of neuronal origin mainly occur within this frequency range (Biswal et al., 1995; Cordes et al., 2001). No filtering was applied to the parcel-averaged EEG-ESI signals.

The dFC was estimated by means of a sliding window correlation approach using a window length of 42 s (21 TRs) with a step of 6 s (3 TRs) for the fMRI data, and a window length of 40 s (40 time points) with a step of 5 s (5 time points) for the EEG-ESI data. Such window lengths were selected based on a recent meta-analysis revealing that physiologically meaningful, and statistically validated dFC fluctuations can be detected on the fMRI when using a window length between 30 and 60 s (Preti et al., 2017). Additionally, our previous study focused on the detection of epileptic dFC states from simultaneous EEG-fMRI data also showed the ability to detect epileptic dFC states irrespective of the window length within the abovementioned interval (Abreu et al., 2019). With this combination of parameters, comparable properties are expected to be captured on fMRI and EEG-ESI dFC data, while guaranteeing that all points at the end of the dFC data are considered when building the respective last sliding windows. The pairwise Pearson correlation coefficient was computed for all pairs of ROI-averaged BOLD-fMRI and EEG-ESI signals for each sliding window. The final dFC matrix was obtained by extracting the upper triangular part of each correlation matrix, vectorising it and subtracting the static FC for each participant (average across all participant-specific windows), yielding a matrix **C**_p_ = [**c**_1_,…,**c**_T_] ∈ *ℝ*^M×T^, where *M* = (*R*^2^−*R*)/2 with *R* denoting the number of ROIs and *T* the number of windows. The group matrix **C** was obtained by concatenating in time the vectorised, static FC-subtracted **C**_p_ matrices of each participant.

##### Identification of dFC brain states

In our previous study, we comprehensively compared the performance of several methods (including the most commonly used *k*-means clustering and principal component analysis) with distinct degrees of sparsity in time, at identifying dFC states associated with epilepsy (Abreu et al., 2019). We found that an *l*_1_-norm regularized dictionary learning (DL) approach yielded the best results. The proposed approach can be formulated as the matrix factorization problem **C** = **DA**, where **D** = [**d**_1_,…,**d**_k_] ∈ *ℝ*^M×k^, and **A** = [**a**_1_,…,**a**_T_] ∈ *ℝ*^k×T^ represent the correlation matrices and associated weight time-courses of each dFC state, respectively; and *k* is the number of dFC states. These are estimated by solving the optimization problem given by:

(2) $$\arg\,\min_{D,A}{||\text{C}-\text{DA}||}_{\text{F}}$$

so that the reconstruction error of **C**, $E={||\text{C}-\text{DA}||}_{\text{F}}^{2}$, is minimized; ||⋅|_|F_ denotes the Frobenius norm of a matrix. The estimation of **D** and **A** was performed using the algorithms implemented in the MATLAB toolbox SPArse Modeling Software (SPAMS, Mairal et al., 2010). The sparsity of the solutions was controlled by a non-negative parameter λ on an *l*_1_-norm regularization framework. The optimal *k* and λ were determined using a 5–5-fold nested cross-validation (Meir-Hasson et al., 2014), whereby the external 5-fold cross-validation divided the data into train and test sets. For each train set (or external fold), an additional inner 5-fold cross-validation was used, from which a set of optimal *k* and λ was determined for each external fold by minimizing the Bayesian information criterion (BIC); this metric penalizes model complexity, thus favoring a more parsimonious estimation of dFC states. The most recurrent set of optimal *k* and λ across the external folds was then identified, characterizing the final dictionary learning model. *k* was varied between 5 and 10, in steps of one; and λ was varied across ten values from 1 to 0.1259, in decreasing exponential steps.

##### Matching of EEG-ESI and fMRI dFC brain states

In contrast with the identification of RSNs, whereby templates derived from previous fMRI studies of large populations are available, dFC state templates are yet to be discovered, as this is still a fairly recent research topic (Preti et al., 2017; Thompson, 2018). Thus, the dFC states estimated from the EEG-ESI data can only be cross, rather than independently, validated by the dFC states estimated from the fMRI data. Because the optimal number of dFC states was *k* = 10 for all runs and for both fMRI and EEG-ESI data (although varying the optimal regularization parameter: λ = 1 for the fMRI, the sparsest solution, and λ = 0.25 for the EEG-ESI, corresponding to a solution of intermediate sparsity), a one-to-one match between the dFC states from both datasets was determined by computing the pairwise spatial correlation between the associated correlation matrices **D**, and identifying the dFC state pairs with the highest, and statistically significant correlation (*p* < 0.05, corrected for multiple comparisons using the Bonferroni correction). Similarly to the assignment procedure of RSNs, in cases of non-mutually exclusive pairs of dFC states, the optimal match was determined by randomizing the order of the fMRI dFC states, and then sequentially, and mutually exclusively, matching them to the EEG-ESI dFC states based on their spatial correlation. The match with the highest average spatial correlation across all ten dFC states was then deemed optimal.

Since dFC states are also characterized by weight time-courses **A** representing their contribution to the overall dFC over time, the Pearson correlation between each dFC state time-course and the contrast defined for mapping the FEPN was computed (for the localizer run), in order to verify which, if any, of the fMRI or EEG-ESI dFC states were associated with the expected brain activity at the FEPN.

##### Validation analyses

Because of the lack of an independent validation as the one available for the RSN analyses by the template maps, additional statistical tests were then performed here. First, the statistical significance of the dFC states estimated from both fMRI and EEG-ESI data recorded on each run was assessed by building a data-driven null distribution, following the work described in Leonardi et al. (2013) and Abreu et al. (2019). Specifically, for each run, the fMRI and EEG-ESI **C** matrices were first FFT transformed and, for each connectivity pair, a random, uniformly distributed, phase was added; the inverse FFT was then applied to transform the data back to the time domain. The null distribution consisted of estimating **D** from the phase-randomized **C** matrices using the optimal parameters *k* and λ, computing the reconstruction error $E\,\left(i\right)={||{\text{C}\text{ -}\text{d}}_{\text{i}}\,{\text{a}}_{\text{i}}||}_{\text{F}}^{2}$ of each dictionary **d***_i_* with weight **a***_i_* (*i* =  1,…,*k*), and repeating this procedure 10,000 times. dFC states estimated from the true data with a reconstruction error (calculated as above) below the fifth percentile of the null distribution in *E* were deemed meaningful.

Next, in order to validate the matching of fMRI and EEG-ESI dFC states, the dFC states estimated from the phase-randomized fMRI data were spatially correlated with the dFC states estimated from the true EEG-ESI data, and their statistical significance investigated.

Finally, in order to assess the impact of the window length, we repeated the procedures described above (estimation of dFC brain states and their matching across modalities, and the respective statistical validation) with three different values of the window length within the adequate interval suggested in Preti et al. (2017): 30, 50, and 60 s, corresponding to 15/30, 25/50, and 30/60 time points for the fMRI and EEG-ESI data, respectively.

## Results

### Identification of RSNs

**FIGURE 1:**
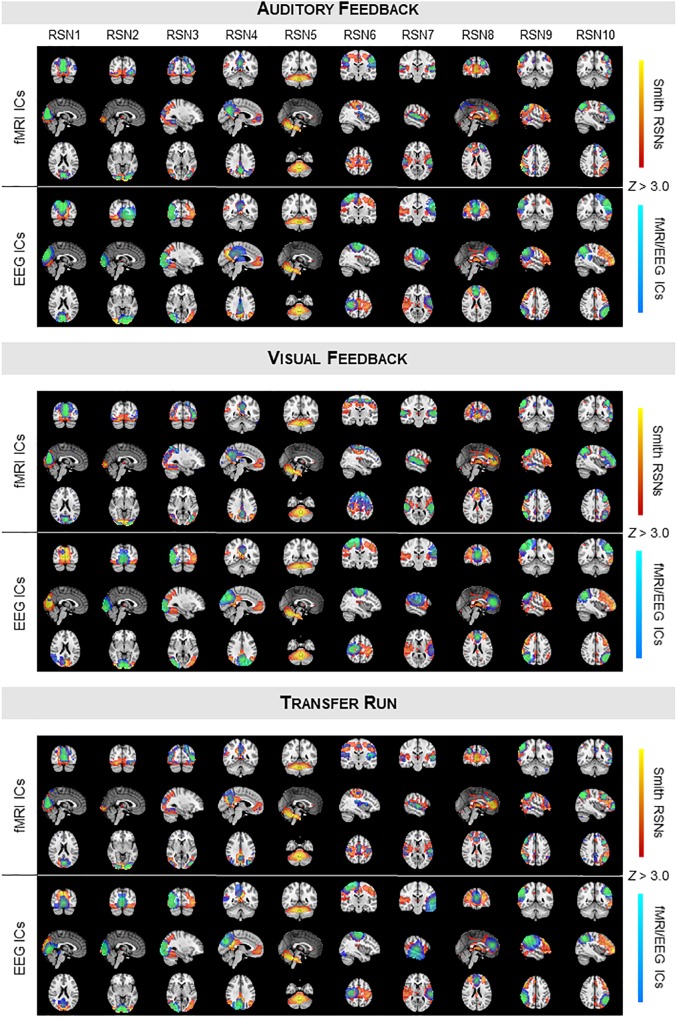
Group RSNs identified on fMRI and EEG-ESI data for the auditory feedback **(top)**, visual feedback **(middle)**, and transfer runs **(bottom)**. For each run, the RSN templates (in red–yellow) from Smith et al. (2009) are superimposed with the fMRI **(top)** and EEG **(middle)** ICs (in blue–light blue) selected for each RSN template, according to their Dice coefficient.

**FIGURE 2:**
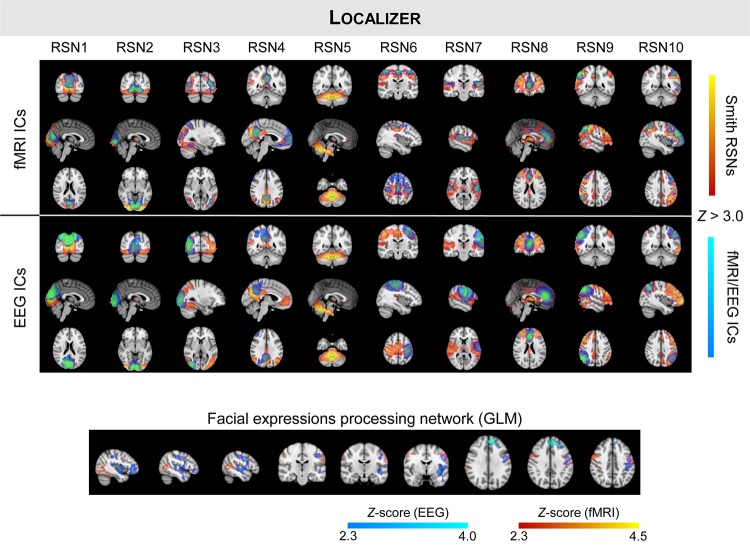
Same as in Figure 1, but showing the group RSNs identified on fMRI and EEG-ESI data for the localizer run. The group activation maps of the task-specific facial expressions processing network using fMRI (in red–yellow) and EEG (in blue–light blue) are also shown at the bottom, with the overlapping regions highlighted in pink.

**FIGURE 3:**
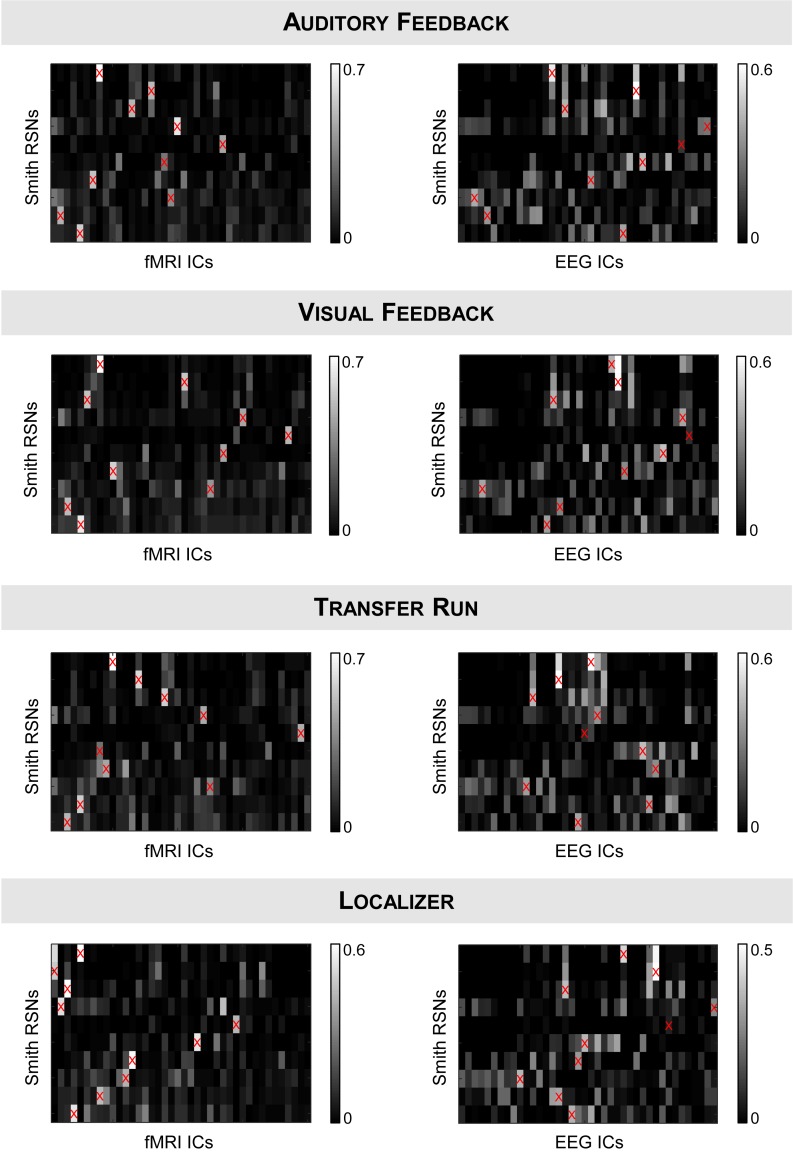
Dice coefficients between all possible combinations of **(left)** fMRI ICs or **(right)** EEG-ESI ICs (*x*-axis) and the RSN templates (*y*-axis) from Smith et al. (2009), for the (from top to bottom) auditory feedback, visual feedback, transfer, and localizer runs. fMRI/EEG-ESI ICs assigned to RSN templates are marked by a red cross.

We started by validating the results from Liu et al. (2017, 2018) in a truly unbiased manner by identifying RSNs on the fMRI and EEG data recorded simultaneously using spatial ICA, and comparing them with the respective templates from Smith et al. (2009); these results are shown in Figure 1 (for the auditory and visual feedback runs, and the transfer run) and Figure 2 (for the localizer run). The selected ICs and the RSN templates show a substantial overlap for all RSNs and imaging techniques, although slightly higher when considering the RSNs identified on the fMRI data, as expected and quantified by the Dice coefficient (values shown in Figure 3, for all possible combinations of fMRI/EEG-ESI ICs and RSN templates, and for all runs). The maximum (*d*_max_) and average ($\bar{d}$) Dice coefficients between fMRI and EEG-ESI RSNs and the RSN templates are depicted in Table 1. Despite the comparable Dice coefficients between the two imaging techniques, with an average of 0.5 and 0.4 for the fMRI and EEG-ESI data across all runs, respectively, it should be noted that RSNs 3 (visual), 6 (motor), and 7 (auditory) were only partially represented on the respective EEG-ESI ICs on all runs, typically including only one of the hemispheres. Additionally, it was not possible to recover RSN 5 (cerebellum) with either fMRI or EEG-ESI data likely because of the limited spatial coverage of this brain region. Noteworthy, however, is the remarkable overall consistency of the RSNs identified on both fMRI and EEG-ESI data across the four runs. The Dice coefficient between the fMRI and EEG-ESI RSNs is also shown in Table 1, with the average values across runs ranging from 0.3 to 0.4, and the maximum values ranging from 0.5 to 0.7. All these values were statistically significant, as they were above the 95th percentile of their respective null distribution.

### Mapping of the FEPN

Next, we mapped the FEPN using a GLM framework on both the fMRI and EEG-ESI data; the group activation maps of all participants is also shown in Figure 2 (bottom). Similarly to what was observed on RSNs 3, 6, and 7, a unilateral (right hemisphere) activation was observed when analyzing the EEG-ESI data, in contrast with the bilateral activation found on the fMRI data. Nonetheless, both maps notably overlap in the left pre-central gyrus. At the posterior superior temporal sulcus (pSTS, visible in the sagittal slices), activations on both fMRI and EEG-ESI were observed, although the latter were found to be located more posteriorly in pSTS, as expected, relatively to the former. Although only present in the EEG-ESI data, frontal activations were also observed.

### Estimation of dFC Brain States

**FIGURE 4:**
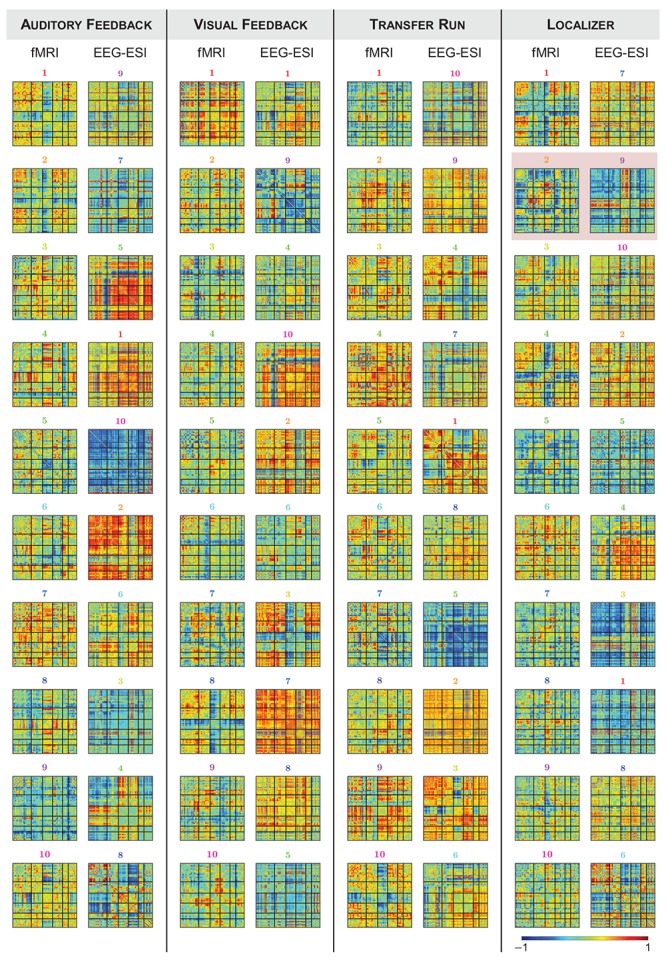
Group dFC states estimated from fMRI and EEG-ESI data for the (from left to right) auditory feedback, visual feedback, transfer, and localizer runs. For each run, the correlation matrices (normalized between –1 and 1, for visualization purposes) of the dFC states estimated from the fMRI data **(left)** and the matched EEG-ESI dFC states **(right)** are shown. For the localizer run, fMRI state #2 and EEG-ESI state #9 are highlighted by the red square, as these two matched dFC states were associated with the activity at the FEPN.

**FIGURE 5:**
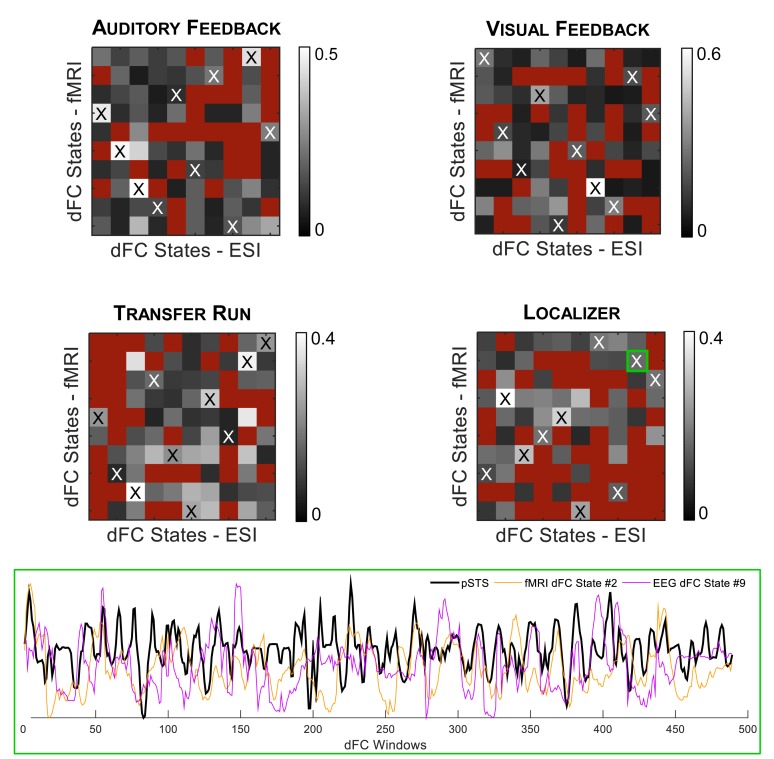
**(Top)** Spatial correlation between all possible combinations of fMRI (*y*-axis) and EEG-ESI (*x*-axis) dFC state correlation matrices, for the auditory feedback, visual feedback, transfer, and localizer runs. Red squares denote non-statistically significant (*p* > 0.05) spatial correlation values. Matched fMRI and EEG-ESI dFC states are marked by the black or white cross. **(Bottom)** For the localizer run, the contribution over time of the matched fMRI state #2 and EEG-ESI state #9 (further highlighted by the green square) is shown (orange and purple traces, respectively), superimposed with the contrast of interest used to map the FEPN (black trace).

Finally, we estimated dFC states from both fMRI and EEG-ESI data using an *l*_1_-norm regularized dictionary learning approach, and identified the optimal match between them. Importantly, all fMRI and EEG-ESI dFC states from all runs survived the statistical validation with phase-randomized data, supporting their physiological meaning. The fMRI dFC states and their matched EEG-ESI dFC states are shown in Figure 4, for all runs. Although it may not be visually clear, a match was obtained between fMRI and EEG-ESI dFC states, quantified by the spatial correlation across the matched dFC states (values shown in Figure 5, for all possible combinations of fMRI and EEG-ESI dFC states, and for all runs). Specifically, the maximum and average spatial correlations (*s*_max_ and $\bar{s}$, respectively) between matched dFC states were *s*_max_ = 0.4, $\bar{s}$ = 0.2 for both the localizer and transfer runs, and *s*_max_ = 0.5, $\bar{s}$ = 0.3 and *s*_max_ = 0.6, $\bar{s}$ = 0.3 for the auditory and visual feedback runs, respectively. This match was validated by statistically assessing the spatial correlation between dFC states estimated from phase-randomized fMRI data, and dFC states estimated from true EEG-ESI data. For all runs, we observed that dFC states did not match in any of the 10,000 randomizations performed, as no statistically significant spatial correlations (*p* > 0.05, corrected for multiple comparisons using the Bonferroni correction) were found between any possible pairs of states, and thus supporting the statistical validity of our findings. Importantly, the spatial correlation values were not substantially affected by the window length, despite a small decrease for larger (>50 s) window lengths; these are shown in Supplementary Table S2. For the additional window lengths and all runs, the estimated dFC states and their match between the fMRI and EEG-ESI also survived the respective statistical tests.

When analyzing the correlation between the weight time-courses of all dFC states and the contrast defined for mapping the FEPN on the localizer run, two fMRI and two EEG-ESI dFC states were found to exhibit a significant (*p* < 0.05, corrected for multiple comparisons using the Bonferroni correction) correlation *r*: states #2 (*r* = 0.22) and #7 (*r* = 0.17) for the fMRI data, and states #4 (*r* = 0.14) and #9 (*r* = 0.19) for the EEG-ESI data; the results are shown in Figure 5, at the bottom. Interestingly, fMRI state #2 is also matched with EEG-ESI state #9 in terms of their spatial correlation, highlighting the consistency of this finding for the localizer run.

## Discussion

Here we comprehensively investigated the extent at which EEG can be used as a tool to identify large-scale functional networks, which are typically detected on fMRI. Importantly, this is the first study to do so in a truly unbiased manner, by comparing the results obtained from fMRI and EEG data acquired simultaneously. Moreover, we investigated for the first time the feasibility of mapping task-specific brain networks, and of estimating the dFC and associated dFC states, with EEG in such conditions.

### EEG and Resting-State Networks

Although the detection of RSNs with EEG was not reported until recently, EEG signatures of RSNs derived from simultaneously acquired fMRI data have already been reported, namely the EEG spectral power averaged across the five well-known EEG rhythms (Goldman et al., 2002; Moosmann et al., 2003; Laufs et al., 2006; Scheeringa et al., 2008). By including the associated time-courses in a regression analysis of BOLD signals representative of the RSNs, the contribution of each EEG rhythm, and its interaction with the remaining rhythms, was found to be specific for each RSN (Mantini et al., 2007; de Munck et al., 2009). Because resting-state EEG is also known to exhibit spontaneous fluctuations that can be described by a limited number of scalp topographies of electrical potentials that remain stable for short (∼100 ms) periods of time (EEG microstates; Michel and Koenig, 2018), it was hypothesized that RSNs were reflected on both EEG and fMRI. Despite their methodological differences, a few studies have confirmed such hypothesis (Britz et al., 2010; Musso et al., 2010; Yuan et al., 2012; Bréchet et al., 2019; Hunyadi et al., 2019), with one study even establishing a one-to-one relationship between EEG microstates and fMRI RSNs (Britz et al., 2010), which, however, has been widely debated (Michel and Koenig, 2018).

Based on these findings, it is therefore expected that RSNs are also explicitly reflected on the EEG. This was first suggested by previous studies showing the ability of functional connectomes (covariance matrices of signals averaged across gray matter parceled according to an atlas) extracted from source-reconstructed EEG data across each EEG rhythm, to predict functional connectomes derived from simultaneously acquired fMRI data (Deligianni et al., 2014), and more recently to predict anatomical connectomes derived from diffusion MRI data as well (Wirsich et al., 2017). Contradictory results, however, have been reported regarding the relative importance of each EEG rhythm for predicting the fMRI functional connectomes. Moreover, these studies were focused on functional connectomes and their derived RSNs, rather than the RSNs more conventionally defined as in Smith et al. (2009).

Such RSNs were first detected on EEG recordings by decomposing, with spatial ICA and at the subject level, the temporally concatenated power of the source-reconstructed EEG data across each rhythm, followed by a clustering step on the resulting ICs from all subjects to identify group-level RSNs (Sockeel et al., 2016). These were then compared with RSNs derived from the fMRI data, with only a sub-set of RSNs presenting a clear overlap between imaging techniques. Considering instead the broadband, rather than band-specific, power of the EEG sources (Liu et al., 2017) also suggested the identification of RSNs on the EEG. Although these findings were validated by RSNs derived from fMRI data, the ground truth considered was not ideal as the two signals were not acquired simultaneously, and therefore do not account for the well-known inter-subject and inter-session variability of the recordings (Smith et al., 2005; Corsi-Cabrera et al., 2007). More importantly, because RSNs result from the temporal coherency of spontaneous activity, cross-validating results from two imaging techniques requires their concurrent acquisition (Abreu et al., 2018). Besides comparing the RSNs obtained from fMRI and EEG data acquired simultaneously, here we considered as ground truth the RSN templates from Smith et al. (2009), which are commonly used in the literature to inform the identification of RSNs. In this way, an independent validation could be performed; one limitation, however, relates with the nature of such templates, as they were derived from fMRI data recorded from a large population of healthy subjects, and therefore may positively bias the amount of overlap (here quantified by the Dice coefficient) between the fMRI RSNs and their templates, relatively to the RSNs derived from the EEG. Our results are in line with this observation, as the maximum and average Dice coefficient across RSNs were systematically higher for the fMRI than those for the EEG, in all four runs. Nonetheless, comparable Dice coefficients were observed between the two imaging techniques; it should be noted, however, that fMRI ICs typically exhibited a notable overlap with a single RSN template, rather than having multiple ICs competing for the RSN template, as observed with some EEG ICs. Such apparent higher specificity of the fMRI data may again be partially explained by the RSN templates being derived from fMRI data. Because the RSNs were obtained from simultaneous EEG-fMRI data, our results further support the existence of EEG correlates of RSNs. Similarly to the fMRI-derived RSN templates, by applying the methodology here proposed to a larger set of EEG-fMRI recordings, EEG-based RSN templates could in principle be obtained, and potentially be used to investigate differences from the fMRI-derived RSN templates.

### Continuous Source Imaging of EEG Acquired Simultaneously With fMRI

In the study of Liu et al. (2017), continuous EEG source imaging (cESI) was performed under optimal conditions, from high-density (256 channels) recordings outside the MR scanner. Despite the potential loss in data quality, the feasibility of performing cESI on EEG data acquired simultaneously with fMRI has already been demonstrated (Groening et al., 2009; Vulliemoz et al., 2009, 2010a,b; Siniatchkin et al., 2010), particularly using EEG caps with a conventional spatial coverage (32 or 64 channels). In fact, simulation studies showed that an almost perfect source reconstruction can be obtained with only 68 channels, reaching a plateau at 100 channels (Michel et al., 2004). Since several processing steps are carried out when performing cESI, their impact on the detection of RSNs with EEG was systematically investigated by Liu et al. (2018), with the electrode density being the most relevant factor, followed by the head model and source localization algorithm chosen. Using the recommended setup and processing pipeline, Liu et al. (2018) reported an average correlation between fMRI and EEG RSNs of 0.6. Here, we used the same processing pipeline, but applied to 64-channel EEG data acquired simultaneously with fMRI, with a maximum/average Dice coefficient of 0.5/0.4 (averaged across runs). These slightly poorer results may partially be explained by the potential loss in data quality due to the presence of MR-induced artifact residuals; nonetheless, our results demonstrate the feasibility of using cESI applied to low-density EEG data acquired simultaneously with fMRI, for detecting RSNs.

### Mapping of Task-Specific Brain Networks With EEG

We used EEG for mapping a task-specific brain network: specifically, we attempted to map the FEPN from the fMRI and EEG recordings of the localizer run using a GLM framework. Although uncommon, such framework has been previously applied to source-reconstructed M/EEG data: the first studies were focused on quantifying the contribution of event-related (Brookes et al., 2004) or band-limited frequency power (Trujillo-Barreto et al., 2008) waveforms associated with a specific activity of interest. By adopting this model-based analysis of the EEG, the generators underlying such activity of interest can in principle be isolated from the remaining ones. Similarly to our study, previous reports have also used GLM to analyze broadband EEG data, for better characterizing the impulse response function associated with different types of visual stimuli (Gonçalves et al., 2014), or for more accurately localizing the generators of EEG microstates (Custo et al., 2014). These studies and their promising results motivated our chosen approach to model EEG-ESI data with the task-specific expected response function, with the additional benefit of providing an activation map directly comparable with that from the conventional GLM analysis of the fMRI data.

Despite the small overall overlap (Dice coefficient of 0.1), the group activation maps of each imaging technique overlapped at the left pre-central gyrus, which is known to be involved in the processing of face expressions (Fox et al., 2009; Radua et al., 2010). Although non-overlapping, relevant fMRI and EEG-ESI activations were found at the postcentral sulcus and the posterior superior temporal sulcus (pSTS), the latter being the anchor of the FEPN (Srinivasan et al., 2016; Wang et al., 2016). The concordant activations at (or close to) pSTS are in agreement with our previous work (Direito et al., 2019), and also with a study specifically focused on the functional segmentation of the STS (Deen et al., 2015). The latter showed that among several social perception and cognition tasks, the participants consistently responded to the perception of faces mainly at pSTS, highlighting the relevance of these activated brain regions. Interestingly, the EEG-ESI data also exhibited frontal activations, which have been shown to play a role in this cognitive task (Kesler-West et al., 2001); however, this result cannot be truly validated as such activations were not present in the fMRI data.

In contrast with the bilateral fMRI activation map, which is consistent with the results from Deen et al. (2015) and Direito et al. (2019), an unexpected lateralization of the FEPN was observed when derived from the EEG data. Such lateralization was also observed on some of the RSNs, where bilateral RSN templates only partially overlapped with unilateral EEG RSNs. Importantly, this does not result from a systematic limitation of cESI, as other bilateral RSN templates were fully recovered by the EEG. Nonetheless, several limitations of ESI have been acknowledged, particularly the non-uniqueness of the inverse problem. This challenge is only partially overcome by making assumptions about the neuronal sources in order to constrain the solution space, which would otherwise be infinite. Furthermore, ESI solutions are strongly biased toward cortical (and focal) sources of electrical activity, because of the lack of sensitivity of reconstruction algorithms with respect to deep gray matter sources, invariably tending to shift solutions to the cortical surface (Michel et al., 2004; Michel and Murray, 2012). Notwithstanding these limitations, we verified consistently overlapping maps for most of the RSNs and the task-specific FEPN, enforcing the feasibility of this approach.

### Dynamic Functional Connectivity and Brain States

Although unreported so far, the match between dFC states derived from fMRI and EEG data found in the present study was somewhat expected, considering that a number of studies have already found EEG correlates of dFC fluctuations and brain states measured with simultaneous fMRI (Tagliazucchi and Laufs, 2015), motivated by the yet unclear physiological underpinnings of dFC (Thompson, 2018). These studies were mainly focused on healthy subjects (Chang and Glover, 2010; Allen et al., 2017) and epilepsy patients (Laufs et al., 2014; Lopes et al., 2014; Preti et al., 2014; Omidvarnia et al., 2017; Abreu et al., 2019). Interestingly, when comparing the contrast of interest for mapping the FEPN with the contribution over time of each fMRI and EEG dFC state, we found that the contribution of two matched dFC states based on their spatial correlation were significantly correlated with the FEPN contrast. The identification of fMRI dFC states specifically associated with a given activity of interest extracted from the EEG had already been suggested (Abreu et al., 2019). While this study shows the potential of fMRI to capture dFC fluctuations associated with specific brain activities, our finding extends it, and specifically suggests that fluctuations in the functional connectivity of the FEPN can be captured by both fMRI and EEG, thus supporting the physiological meaning of the also statistically validated match found between fMRI and EEG dFC states. Our results thus further evidence the existence of EEG correlates of dFC, and open new lines of research where dFC fluctuations of large-scale functional networks can be investigated with EEG, a technique that more directly measures brain activity when compared with fMRI.

### Limitations

In this multimodal study, a small sample size was considered, which inevitably hinders our conclusions to be generalized to future studies applying the proposed methodology. Nonetheless, given the consistency of our results regarding the detection of RSNs, the mapping of the task-related FEPN, and the identification of matched dFC states across modalities, particularly those associated with the FEPN, we believe that this study provides a strong proof-of-concept on the use of EEG as a brain imaging tool.

Another important aspect of our study is that the participants were performing a neurofeedback (NF) task. This relates to our additional goal of testing the transfer of an already validated fMRI NF intervention to an EEG setup, with the purpose of generalizing and disseminating such intervention. This motivates this study’s investigation on the possibility of mapping a task network of interest (the face expressions processing network, FEPN) with EEG. Despite some reported differences between co-activation networks and RSNs (Di et al., 2013), it has been shown that RSNs can also be accurately identified on fMRI data collected from participants performing tasks in general (Cole et al., 2016). This has thus motivated our study to address both the intrinsic and task-related connectivity aspects from a static and dynamic point of view. NF tasks, however, are known to modulate the strength of intra- and inter-network connections, which may influence the functional organization of RSNs (Sitaram et al., 2017). Importantly, the NF task used in this study was tailored to modulate the percent signal change of the BOLD signal measured at a limited and well-defined brain region in pSTS, rather than the connectivity strength of specific RSNs (Direito et al., 2019). Moreover, assuming that this NF task is nonetheless modulating the RSNs, it has also been shown that NF is able to induce the desired changes on both EEG and fMRI when performing real-time simultaneous EEG-fMRI NF (Zich et al., 2015; Zotev et al., 2016). It is then expected that potential modulations on the RSNs and/or dFC states will be reflected on both EEG and fMRI data, and thus in principle not confounding our results. Naturally, this could only be confirmed in future studies applying the proposed methodologies to resting-state data.

## Conclusion

In this study, we validated in a truly unbiased manner the existence of RSNs reflected on both fMRI and EEG data, while also supporting the feasibility of continuous electrical source imaging to low-density EEG data acquired simultaneously with fMRI. We also showed that EEG can be used for mapping task-specific networks (particularly the facial expression processing network, FEPN), as well as to study the dynamics of functional networks, and extract their representative dFC states. Importantly, we also determined that fluctuations in the functional connectivity of the FEPN can be captured on both fMRI and EEG. Additionally, our results support the emerging literature on EEG correlates of (dynamic) functional connectivity measured with fMRI, and therefore provide novel insights into the coupling mechanisms underlying the two imaging techniques. Our analyses push the limits of EEG toward being used as a brain imaging tool, allowing researchers and clinics to more efficiently leverage the high temporal resolution, low cost, portability and ease of use that characterize the EEG.

## Data Availability Statement

The datasets generated for this study are available on request to the corresponding author.

## Ethics Statement

The studies involving human participants were reviewed and approved by Ethics Commission of the Faculty of Medicine of the University of Coimbra. The patients/participants provided their written informed consent to participate in this study.

## Author Contributions

RA was responsible for performing all the data analyses and writing the manuscript. MS acquired all the data, and was responsible for designing and coding the experimental protocol, and reviewed the manuscript. MC-B supervised this study, and reviewed the manuscript.

## Conflict of Interest

The authors declare that the research was conducted in the absence of any commercial or financial relationships that could be construed as a potential conflict of interest.

## Footnotes

1 https://fsl.fmrib.ox.ac.uk/fsl/fslwiki/MELODIC
