# Supplementary material for: Pushing the Limits of EEG: Estimation of Large-Scale Functional Brain Networks and Their Dynamics Validated by Simultaneous fMRI
Source: Front Neurosci. 2020 Apr 16;14:323. doi: 10.3389/fnins.2020.00323 (PMC7177188; doi:10.3389/fnins.2020.00323)
Supplement: Supplementary file 1 [file Data_Sheet_1.PDF]

Supplementary Material

1 Experimental protocol for the neurofeedback runs

The session consisted of three EEG-fMRI neurofeedback (NF) runs of alternated up and down regulation, using mental imagery of facial expressions as strategy to modulate the activity of the target brain region, the posterior superior temporal sulcus (pSTS). In the first two runs, visual or auditory feedback was provided to the participant, while in the third run no feedback was provided. The runs consisted of interleaved blocks of up- and down-regulation with 24 seconds each (Figure S1). The technical aspects of the NF MRI acquisition were identical to the localizer run. Each run had a total duration of 10 minutes, corresponding to a total of 300 volumes.

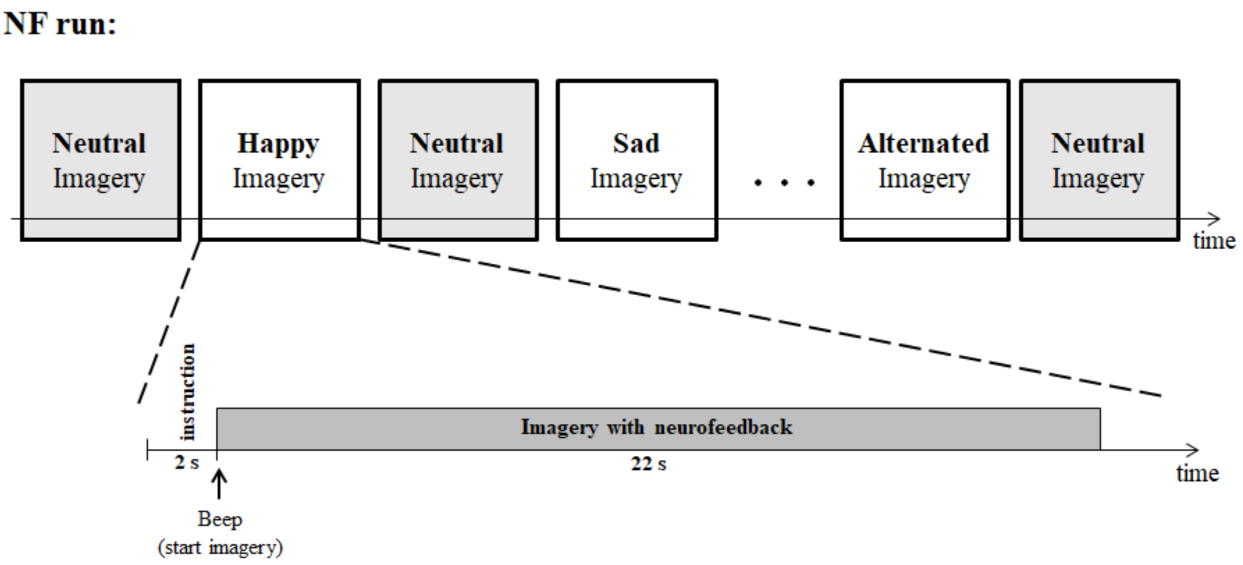

Figure S1: Experimental protocol for the neurofeedback runs (for both the visual, auditory and transfer runs).

For the visual feedback, the percent signal change (PSC) of the BOLD signal measured at the pSTS region of the face expressions processing network (FEPN, mapped according to the localizer run) was calculated by normalizing the average BOLD signal amplitude at each volume by a baseline defined as the average BOLD signal across sliding windows comprising the last 10 volumes of the Neutral condition. The PSC was then normalized and segmented into 15 levels between 0 and 1.5%. The final level was used to define the intensity of the morph of the facial expression of the virtual avatar that was presented as feedback to the participant. For the first 6 seconds of each block, no feedback was provided (avatar maintained the morphing level from the end of the previous block) in order to compensate for the hemodynamic delay.

The auditory feedback was computed based on the local changes of the BOLD signal, instead of its variation in relation to a baseline window; this was defined as the slope of the BOLD signal estimated from the last 3 volumes. If positive, a high-pitch sound was provided as feedback to the participant, informing him/her that the BOLD signal amplitude at the FEPN was increasing, as desired. Conversely, if the slope was negative, a low-pitch sound was provided. To reduce the feedback clutter of the experiment, the sounds were played only three times at each block, at volumes 5, 8 and 11. The avatar was only displayed during the instruction phase; a black screen was displayed for the remaining part of the block.

2 Additional information on the forward model (EEG source reconstruction)

Tissue	Conductivity (S/m)	# FEM elements
Skin	0.4348	436047
Eyes	0.5000	13195
Muscle	0.1000	616533
Fat	0.0400	163140
Spongy bone	0.0400	319347
Compact bone	0.0063	931291
Cortical gray matter	0.3333	666778
Cerebellar gray matter	0.2564	100049
Cortical white matter	0.1429	323976
Cerebellar white matter	0.1099	22544
Cerebrospinal fluid	1.5385	579890
Brainstem	0.5000	26539

Table S1: Conductivity values of the 12 tissue class segmented according to the MIDA tissue model, considered for computing a realistic head model for EEG source reconstruction.

3 dFC state analysis varying the window length

The dFC state analyses were repeated with three different additional values of the window length within the adequate interval suggested in (Preti et al., 2017): 30, 50 and 60 s, corresponding to 15 / 30, 25 / 50 and 30 / 60 time points for the fMRI and EEG-ESI data, respectively.

The maximum and average spatial correlation values (s_{max} and $\bar{s}$, respectively) between the 10 matched dFC states for each window length considered are depicted in Table S2.

Functional runs	Window length [s]							
	30		40		50		60	
	s_{max}	$\bar{s}$	s_{max}	$\bar{s}$	s_{max}	$\bar{s}$	s_{max}	$\bar{s}$
Auditory Feedback	0.6	0.3	0.5	0.3	0.4	0.2	0.5	0.2
Visual Feedback	0.6	0.3	0.6	0.3	0.4	0.3	0.4	0.2
Transfer Run	0.4	0.3	0.4	0.2	0.3	0.2	0.4	0.2
Localizer	0.3	0.2	0.3	0.2	0.3	0.2	0.3	0.2

Table S2: Maximum and average spatial correlation values (s_{max} and $\bar{s}$, respectively) between matched dFC states for each window length and functional run.
